# Analysis of the experimental absorption spectrum of the rabbit lung and identification of its components

**DOI:** 10.1002/jbio.202300494

**Published:** 2024-03-07

**Authors:** Maria R. Pinheiro, Valery V. Tuchin, Luís M. Oliveira

**Affiliations:** ^1^ Institute for Systems and Computer Engineering, Technology and Science (INESC TEC) Porto Portugal; ^2^ Department of Electrical and Computer Engineering Porto University – Faculty of Engineering Porto Portugal; ^3^ Science Medical Center Saratov State University Saratov Russian Federation; ^4^ Laboratory of Laser Molecular Imaging and Machine Learning Tomsk State University Tomsk Russian Federation; ^5^ Physics Department Polytechnic Institute of Porto – School of Engineering Porto Portugal

**Keywords:** absorption spectrum reconstruction, lung absorption spectrum, photon diffusion approximation, pigment accumulation, spectral physiological information, tissue component identification

## Abstract

The broadband absorption coefficient spectrum of the rabbit lung presents some particular characteristics that allow the identification of the chromophores in this tissue. By performing a weighted combination of the absorption spectra of water, hemoglobin, DNA, proteins and the pigments melanin and lipofuscin, it was possible to obtain a good match to the experimental absorption spectrum of the lung. Such reconstruction provided reasonable information about the contents of the tissue components in the lung tissue, and allowed to identify a similar accumulation of melanin and lipofuscin.
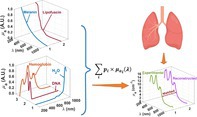

## Graphical abstract

The broadband absorption coefficient spectrum of the rabbit lung was reconstructed from the absorption spectra of tissue components. The similar accumulation of melanin and lipofuscin was retrieved from the broadband baseline in the absorption coefficient spectrum, and the calculation of the absorption fold ratios for proteins, DNA and hemoglobin provided good results. The method used is innovative and can be improved to allow the quantification of tissue components concentrations directly.

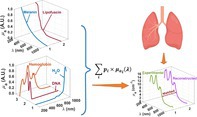

## 1 INTRODUCTION

Soft biological tissues are heterogeneous materials that contain various components, such as lipids, DNA, proteins, blood, water, pigments, and others. Each of these components presents characteristic absorption bands that can be located in narrow or broadband spectral ranges. The broadband absorption coefficient spectrum, *μ*_*a*_(*λ*), of any tissue allows to identify the tissue components and may provide information about the contents of those components. As previously reported [1], the *μ*_*a*_(*λ*) of a tissue can be described as a weighted sum of the absorption spectra of the tissue components, *μ*_*ai*_(*λ*):

(1) $$\mu_{a}\left(\lambda \right)=\sum_{i}p_{i}\times \mu_{\mathrm{ai}}\left(\lambda \right),$$

with *p*_i_ representing the weight (or contribution) of the absorption spectrum of the *i* component to the global absorption spectrum of the tissue.

Considering the availability of the *μ*_*ai*_(*λ*) of various tissue components, it becomes interesting to digitally reconstruct the experimental broadband *μ*_*a*_(*λ*) of different tissues, so that physiological or diagnostic information can be accessed through optical methods [2].

The *μ*_*a*_(*λ*), as well as the other spectral optical properties of tissues, can be obtained for a broad spectral range using different methods, which are simply classified into two main groups: the one that involves inverse computer simulations and the one that relies on direct calculations from experimental data [2]. The methods in the first group use a simulation algorithm that solves the radiation transfer equation, while the methods in the second group are based on direct calculations from experimental measurements using the photon diffusion approximation [2].

Considering the first group of methods, some computer codes are available for immediate use, or that can be easily adapted, to estimate the optical properties of biological tissues. Such codes are based on the algorithms of Kubelka–Munk, Monte Carlo, and Adding‐Doubling [2, 3, 4, 5, 6, 7, 8]. In these simulations, using data from experimental optical measurements that were acquired from the tissue under study, the algorithm estimates a set of two or three optical properties at a particular wavelength (*λ*). Usually, the input data to these simulations are the refractive index (RI), the total transmittance (*T*_*t*_), the total reflectance (*R*_*t*_), and the collimated transmittance (*T*_*c*_) of the tissue at a single *λ* [5]. Since each set of the estimated optical properties refers to this single *λ*, and with the objective of obtaining the broadband spectral optical properties of the tissue, individual simulations at discrete *λ* values within the desired spectral range are necessary. After terminating those simulations, the discrete estimated data for each optical property can be interpolated with the appropriate curves [7]. Such a procedure to estimate the spectral optical properties is time consuming and computer demanding, preventing a fast determination of the broadband spectra of tissue optical properties [9].

Various studies have been made using the simulation methods in the past. One of such studies was conducted with the inverse Adding‐Doubling (IAD) simulations to estimate the spectral optical properties of healthy and cancerous (meningioma, pituitary adenoma, schwannoma, low‐ and high‐grade glioma) tissues of the human brain between 400 and 1800 nm [10]. The estimated *μ*_*a*_(*λ*) was similar for all tissues, but the spectral reduced scattering coefficient, *μ*'_*s*_(*λ*), showed significant differences between the healthy and tumor tissues, which can be used for diagnostics. Using 22 ex vivo human skin samples, *Troy & Thennadil* performed another study, also based on the IAD simulations, to estimate the skin's spectral optical properties between 1000 and 2200 nm, where from *μ*_*a*_(*λ*) it was possible to identify the presence of water and lipids [11]. The study reported by Bashkatov et al. used also the IAD simulations to estimate the *μ*_*a*_(*λ*), the *μ*'_*s*_(*λ*), and the spectral optical penetration depth in diffusion approximation (*δ*(*λ*)) of human skin, subcutaneous and mucous tissues in the 400–2000 nm spectral range [12]. The same group published two other interesting studies where the inverse simulations were used to estimate the spectral optical properties for peritoneal tissues between 350 and 2500 nm [13], and for human stomach mucosa between 400 and 2000 nm [14]. Considering the *μ*_*a*_(*λ*) of the tissues that were obtained in these studies, they contain characteristic absorption bands, and, consequently, this spectral property may be considered as an identity card for each tissue.

Considering the group of methods that involve direct calculations of tissue's optical properties from experimental data, they present the benefits of being faster and less computer demanding [9]. Using such methods, if spectral measurements of *T*_*t*_, *R*_*t*_, and *T*_*c*_ are available for the tissue under study in the desired spectral range, the calculation of most of the optical properties can be made through equations that were developed based on the photon diffusion approximation [9, 15, 16]. The use of such methods in recent studies has allowed to retrieve valuable physiological and diagnostic information from the *μ*_*a*_(*λ*) of healthy and diseased tissues [15, 16, 17], which can be used in future diagnostic protocols. As previously reported, the contents of blood, proteins and DNA are differentiated between healthy and diseased tissues [2, 18], and can be retrieved from the analysis of the experimental *μ*_*a*_ spectra of biological tissues. Furthermore, it is known that tissues accumulate pigments, such as melanin and lipofuscin, during the aging process [2]. The fast and direct calculation of the *μ*_*a*_(*λ*) of rabbit brain cortex allowed Gonçalves et al. to correlate an excessive content of melanin with neurodegenerative processes that occur in the brain [15]. In this and other recent studies [16, 17], the *μ*_*a*_(*λ*) of different healthy and diseased tissues was directly calculated from the measured *T*_*t*_ and *R*_*t*_ spectra, according to the following Relation (2), [15, 16, 17, 19]:

(2) $$\mu_{a}\left(\lambda \right)=\frac{1-\left[T_{t}\left(\lambda \right)+R_{t}\left(\lambda \right)\right]}{d},$$

where *d* represents the thickness of the tissue samples under study. Using this calculation method, a study by Carvalho et al. showed that the analysis of the *μ*_*a*_(*λ*) of human colorectal mucosa tissues allows one to identify a higher lipofuscin content in the diseased (adenocarcinoma) mucosa [19]. In the case of the human kidney, Botelho et al. used the same method to evaluate the melanin and lipofuscin contents from the calculated *μ*_*a*_(*λ*) both for healthy and diseased (chromophobe renal cell carcinoma [CRCC]) tissue samples [17]. From the results obtained in that study, it appeared that the melanin in the healthy kidney samples has converted into lipofuscin in the CRCC kidney samples. Since such conversion between pigments had not been previously reported, the authors concluded that the development of CRCC in the human kidney was associated with the formation of melanolipofuscin granules, which consists of melanin surrounded by a lipofuscin shell.

It is important to stress that both approaches to obtain the spectral optical properties of biological tissues require *T*_*t*_ and *T*_*c*_ data, which are easily obtained from excised tissue samples. These measurements are impracticable to perform in vivo and if they were feasible, they should be considered highly invasive. With the objective of evaluating the optical properties of in vivo tissues, machine learning, ML, algorithms, based on non‐invasive or minimally invasive measurements, such as the diffuse reflectance (*R*_*d*_) spectroscopy, can be developed. It should be mentioned that for the validation of such algorithms, the optical properties of the tissue under study need to be first estimated or calculated with any of the methods presented above, which are based on measurements that are acquired from tissue biopsies. In fact, some ML algorithms have already been developed in a few studies. A study developed by *Hokr* and *Bixler* presents a technique where the ML algorithm inverts a Monte Carlo simulation to extract the optical properties from the statistical moments of the spatio‐temporal response of the tissue [20]. A recent review of the application of ML to NIR spectroscopy, focus on the estimation of the *μ*_a_(*λ*) and the use of feature selection algorithms to identify the absorption bands in that spectral range [21]. A study by Zhang et al. reported the development of a non‐segmentation‐based iterative algorithm that is capable of reconstructing the pixel‐wise *μ*_*a*_ map from a photoacoustic tomography image [22]. According to the authors, such algorithm is capable of optimizing the light fluence distribution and consequently the resulting *μ*_*a*_ map. A study from Magnussem et al. resulted in the development of a ML algorithm, based on deep convolutional neural network that recovers pure absorption spectra from highly scatter‐distorted spectra of cells [23]. According to the authors of [21], the use of ML methods to construct the optical properties of in vivo tissues is a highly significant future research direction.

Considering the studies indicated above that use calculations based on the photon diffusion approximation, and the analysis performed on the broadband *μ*_*a*_(*λ*) of tissues that resulted from direct calculations from the spectral measurements, it was possible to obtain valuable physiological and diagnostic information in a fast manner. Consequently, a similar approach can be used to analyze the broadband *μ*_*a*_(*λ*) of other tissues and access physiological and/or diagnostic information directly from spectral measurements. With the objective to contribute to this line of research, the present study consisted of using the diffusion approximation for a fast calculation of the *μ*_*a*_(*λ*) for the rabbit lung. An analysis of this spectrum and its reconstruction from the spectra of certain tissue components allowed to retrieve physiological information for the lung tissue.

## 2 MATERIALS AND METHODS

The spectral measurements performed in this study were acquired from ex vivo lung tissues from rabbits. The protocols adopted in this research follow the Declaration of Helsinki regarding the use of animals in research, and were approved by the Ethics Commission of the Institute for Systems and Computer Engineering, Technology and Science (INESC TEC), as stated in the official document of this Commission from July 6, 2023.

Section 2.1 describes the collection of lung fragments and the preparation of the tissue samples for the spectral measurements. Section 2.2 describes the experimental setups and the measuring protocols used to acquire the *T*_*t*_ and the *R*_*t*_ spectra from the samples, which were used to calculate the *μ*_*a*_ spectra of the lung for further analysis and reconstruction, as described in Section 2.3.

### 2.1 Tissue samples

**FIGURE 1:**
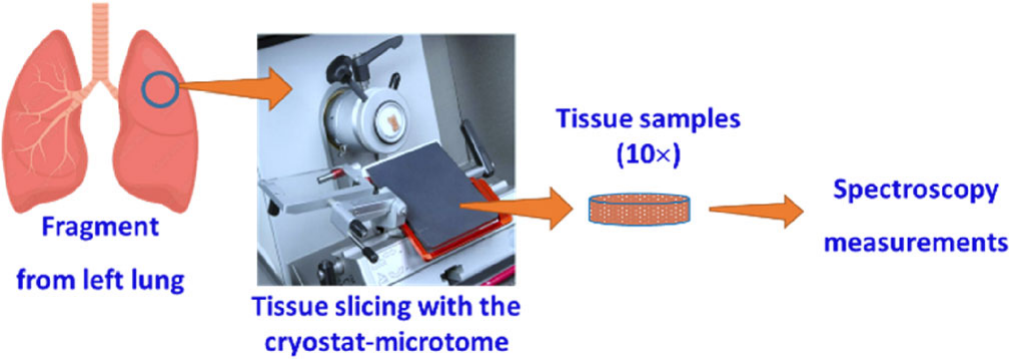
Sequence of steps for sample preparation.

Three adult gray rabbits, all with ~36 months of age, were acquired from the local meat market, immediately after sacrifice. The left lung was retrieved from each animal and preserved at −20°C for 12 h overnight. On the following day, a fragment was dissected from each lung and a cryostat‐microtome from Leica Biosystems (model CM 1850 UV) was used to prepare a total of 10 samples to be used in the spectral measurements. Four samples were prepared from the fragment of the first lung and three others were prepared from each of the fragments collected from the lungs of the other two animals. All the samples had approximate circular form (*ϕ* = 1 cm) and 0.5 mm thickness and they were prepared as they were needed for the spectral measurements. To mimic the hydration of the in vivo lung, before being submitted to the spectral measurements, the samples were kept in saline for 10 min. Figure 1 presents the sequence of steps for the sample collection and preparation.

### 2.2 Spectroscopy measurements

**FIGURE 2:**
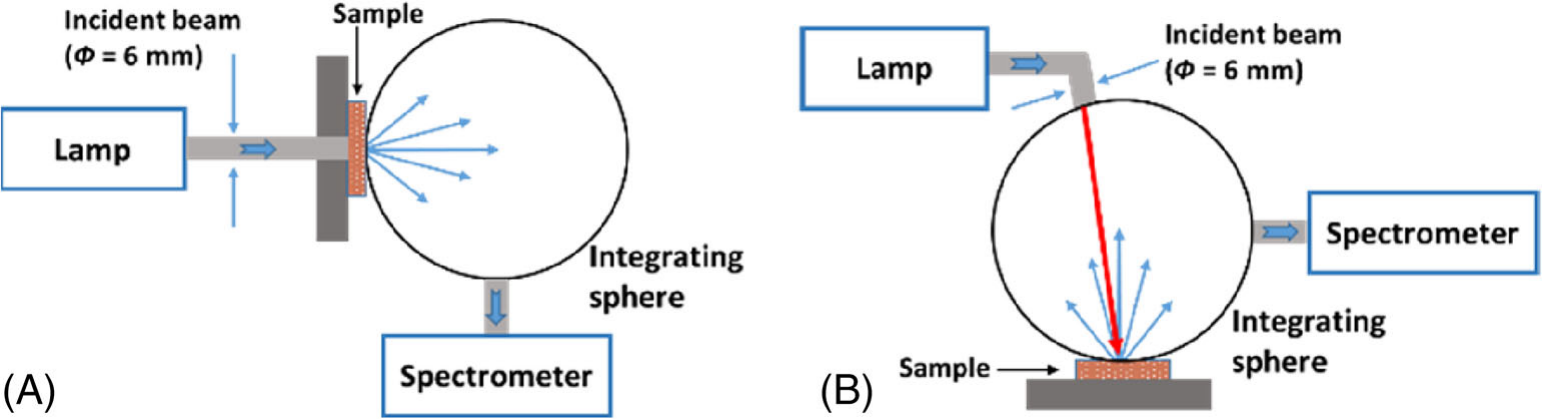
Spectral measurements, showing the *T*_*t*_ setup (A) and the *R*_*t*_ setup (B).

The 10 lung samples were sequentially submitted to two sets of spectral measurements. As represented in Figure 2, each sample was first used to measure its *T*_*t*_(*λ*), and then used to measure its *R*_*t*_(*λ*).

According to the *T*_*t*_ setup presented in Figure 2A, the beam from a broadband and high‐power pulsed Xenon lamp (AvaLight‐XE‐HP from Avantes™) is delivered by an optical fiber cable and collimating lens to irradiate the tissue sample. This collimated beam, with a diameter of 6 mm, crosses the tissue sample into the inside of an integrating sphere (AvaSphere‐50 from Avantes™). The light transmitted by the tissue sample, both collimated and diffuse, is reflected repeatedly in the internal wall of the integrating sphere (integration process) for later collection by another optical fiber cable to be delivered to the spectrometer (AvaSpec‐2048‐USB2 from Avantes™), where the spectrum is registered. In the case of the *R*_*t*_ setup, the equipment is the same that was used in the *T*_*t*_ setup. The difference between the two setups is that for the *R*_*t*_ measurements, the irradiating beam is delivered to the sample through the top side of the integrating sphere, with an inclination of 8° with the sphere's vertical axis. With such an arrangement, the irradiating beam is reflected by the sample to the inside of the sphere to undergo the integration process before being delivered to the spectrometer for spectrum registration. All the *T*_*t*_ and *R*_*t*_ spectra were acquired between 200 and 1000 nm, with a spectral resolution of 1 nm.

After performing all measurements from the 10 lung samples, both with the *T*_*t*_ and *R*_*t*_ setups, the spectra were submitted to calculations to obtain the corresponding experimental *μ*_*a*_ spectra. Such calculations are described in the following sub‐section.

### 2.3 Calculations to obtain the experimental and reconstructed *μ_a_* (*λ*)

Considering both the *T*_*t*_ and *R*_*t*_ spectra measured from each lung sample, the calculation of the corresponding *μ*_*a*_(*λ*), was performed according to Equation (2). In a first attempt to analyze the absorption spectrum of the lung, the mean and standard deviation (SD) of the *μ*_*a*_(*λ*) were obtained for further analysis. The mean *μ*_*a*_(*λ*) of the lung presented various characteristics, such as the occurrence of certain absorption bands that correspond to specific tissue chromophores and a broadband baseline that decreases with increasing *λ*. This last feature was also observed in previous studies and it was associated with the broadband absorption of pigments as a result of the accumulation of melanin and lipofuscin in those tissues [2, 9, 15, 16, 17]. In a first step to analyze the *μ*_*a*_(*λ*) of the lung, such broadband baseline, *μ*_*a*‐baseline_(*λ*), was digitally reconstructed from the normalized absorption spectra of melanin, *μ*_*a*‐Mel_(*λ*), and lipofuscin, *μ*_*a*‐Lip_(*λ*), according to the following Relation (3),

(3) $$\mu_{a-\text{baseline}}\left(\lambda \right)=p_{\mathrm{Mel}}\times \mu_{a-\mathrm{Mel}}\left(\lambda \right)+p_{\mathrm{Lip}}\times \mu_{a-\mathrm{Lip}}\left(\lambda \right).$$

**FIGURE 3:**
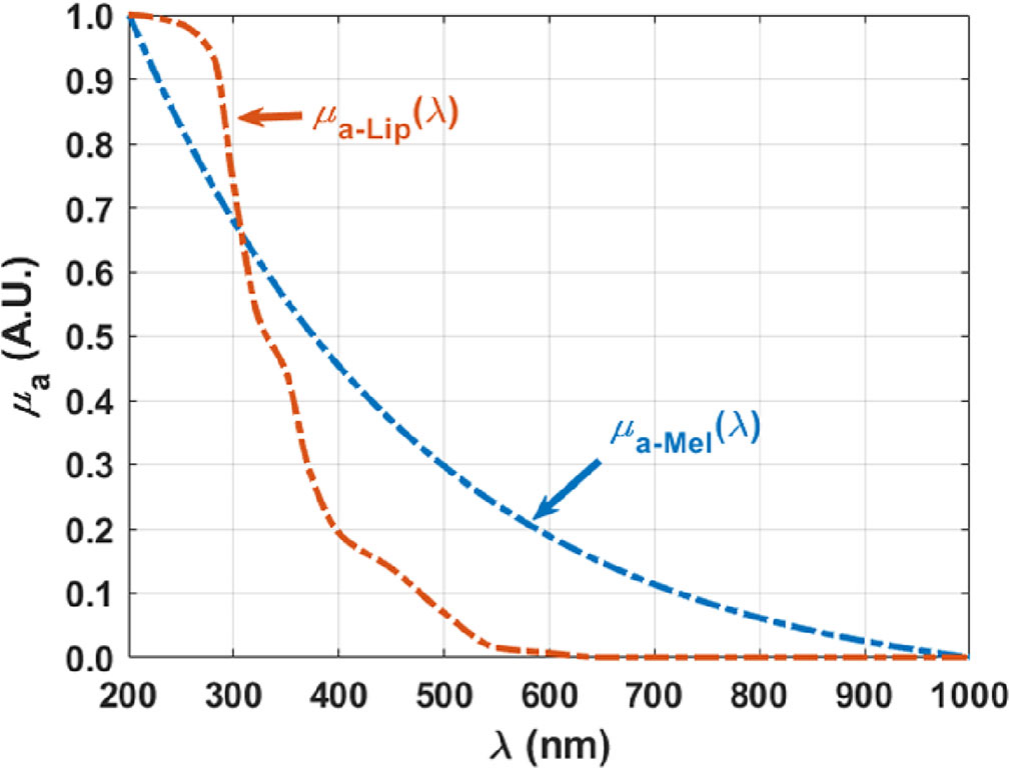
Normalized *μ*_*a*‐Mel_(*λ*) and *μ*_*a*‐Lip_(*λ*) between 200 and 1000 nm. Adapted from Ref. [15], which was published under Creative Commons License, MDPI.

The optimization of the reconstructed baseline allowed to quantify and compare between the contributions of melanin, *p*_Mel_, and of lipofuscin, *p*_Lip_ to infer about their accumulation in the rabbit lung. Figure 3 presents the normalized *μ*_*a*‐Mel_(*λ*) and *μ*_*a*‐Lip_(*λ*) that were used in Equation (3) to reconstruct the *μ*_*a*‐baseline_(*λ*).

After obtaining the reconstructed *μ*_*a*‐baseline_(*λ*), it was subtracted from the mean *μ*_*a*_(*λ*) of the lung for a better analysis of the other tissue components. In previous studies where such a baseline was also observed in the *μ*_*a*_(*λ*) of other tissues, it was verified that the broadband absorption of pigments was camouflaging the real contents of other tissue components, such as DNA and hemoglobin [19]. Only by removing the reconstructed *μ*_*a*‐baseline_(*λ*) from the *μ*_*a*_(*λ*) of such tissues [2], it was possible to calculate accurate absorption fold‐ratios for the other tissue chromophores. The absorption fold‐ratio for a certain chromophore, which provides a reasonable estimate of the chromophore's content in the tissue, is the ratio between the *μ*_*a*_ value at the peak of the absorption band of that chromophore and the baseline. The same procedure was performed for the lung and a comparison between the absorption fold‐ratios for the tissue chromophores, before and after removing the baseline, is made in Section 3.

**FIGURE 4:**
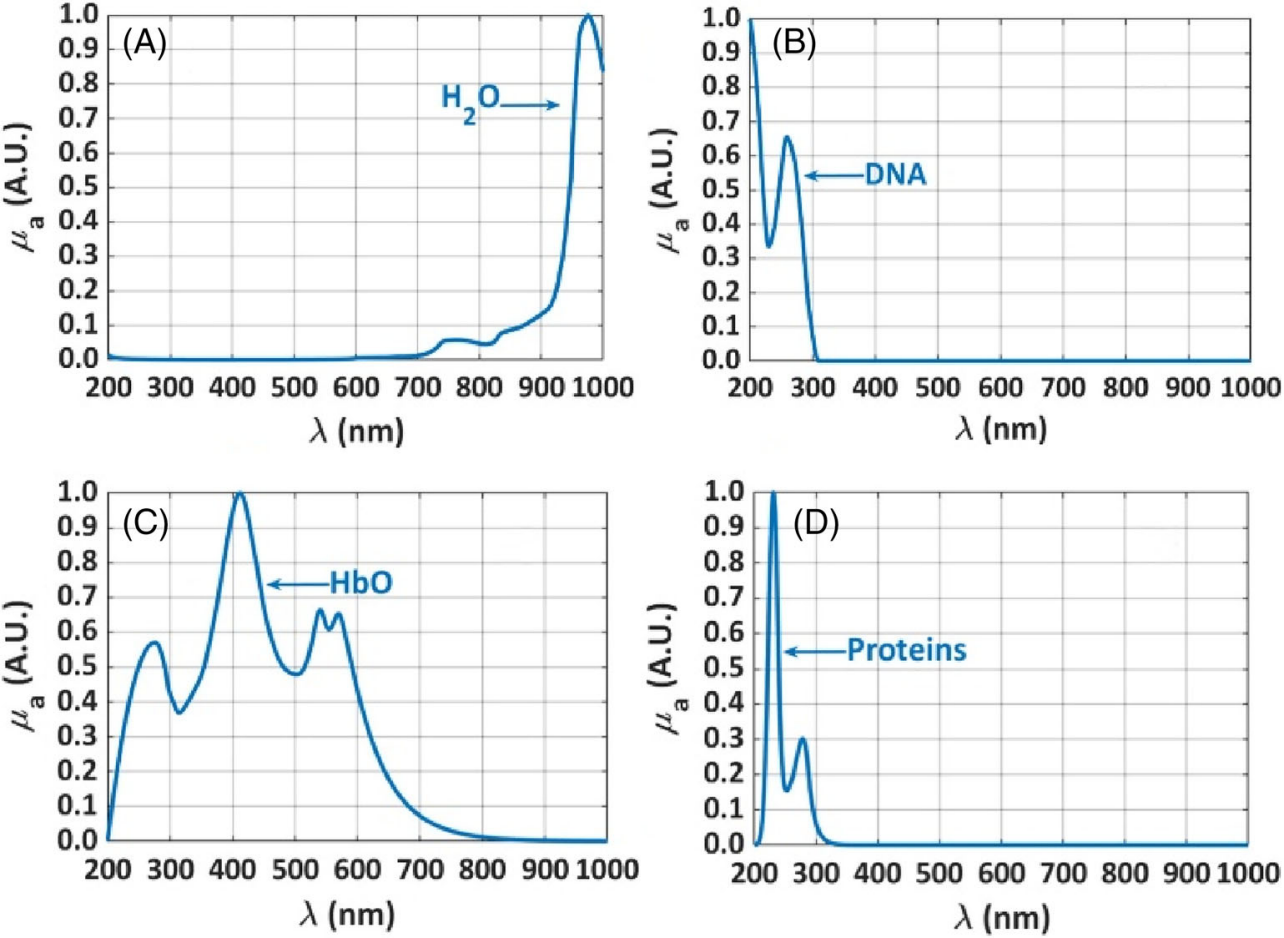
The normalized absorption spectra of water (A), DNA (B), HbO (C), and proteins (D) between 200 and 1000 nm.

In a final attempt to acquire further physiological information from the spectral absorption of the lung, a digital reconstruction of its *μ*_*a*_(*λ*) was made from the absorption spectra of various tissue chromophores. Such reconstruction was made according to Equation (1). When performing this reconstruction of the mean *μ*_*a*_(*λ*) of the lung, in addition to the absorption spectra of melanin and lipofuscin that are presented in Figure 3, the *μ*_*a*_(*λ*) of water (H_2_O) [24, 25, 26], DNA [27], oxygenated‐hemoglobin (HbO) and proteins [28], which in the case of the lung are composed by elastin and collagen [29], were also considered. The spectra of these chromophores are presented in Figure 4, where all graphs were also converted to a normalized scale between 0 and 1 for comparison. In addition to the objective of allowing a better comparison between the *μ*_*ai*_(*λ*) presented in Figures 3 and 4, the conversion of these spectra to such scale, between 0 and 1, was necessary because, due to independent measurement arrangements that originated these spectra, each of them presents different magnitude values. While the maxima observed in the original absorption spectra of melanin, lipofuscin, water, proteins and HbO range between 0.5 and 3 cm^−1^, the maximum observed in the original *μ*_*a*_(*λ*) of DNA reaches values 10^4^× higher. This way, the conversion of these absorption spectra to a similar scale turns the reconstruction of the *μ*_*a*_(*λ*) of the lung easier to perform.

The normalization of each spectrum in Figures 3 and 4 was performed by displacing the entire spectrum to have a minimum equal to zero and then divided by its maximum value. This way, all these spectra are represented in the same scale for a better comparison and to allow an easier reconstruction of the *μ*_*a*_(*λ*) of the rabbit lung.

The *μ*_*a*_(*λ*) of HbO is available in some publications [30, 31], but for a shorter spectral range than the one used in the present study. To obtain the *μ*_*a*_(*λ*) of HbO between 200 and 1000 nm, which is represented in Figure 4C, we have measured it in our lab. To perform this measurement, and since rabbit blood was not available due to ethical reasons, human blood was used as the best second choice. A human volunteer agreed to supply some blood drops, which were diluted in water in different concentrations. The *μ*_*a*_ spectra of the various solutions were measured and the one presented in Figure 4C was the one that provided a better reconstruction of the absorption spectrum of the rabbit lung.

Although the contents of the various blood components may differ a little between human and rabbit blood, they are the same [32]. On the other hand, by mixing human blood with water for our measurements, due to cell lysis that occurs as a result of osmotic stress, the spectrum presented in Figure 4C corresponds to HbO, since there is a great content of dissolved oxygen in water to bind to the released Hb.

It is important to say that the selection of the *μ*_*a*_(*λ*) of HbO for the reconstruction of the absorption spectrum of the lung is adequate. Blood contains various components at the cellular and molecular levels, but among those, Hb and HbO are the ones that present the highest absorption values in the spectral range considered for this study, with at least two orders of magnitude higher than other blood components [31]. Considering the cellular level, blood contains red blood cells (RBC), white blood cells (WBC) and platelets, where, in terms of blood volume their sum occupies about 45%, but WBC and platelets represent less than 1%. The remaining 55% of the blood volume is occupied by the blood plasma [33]. If we consider the molecular level, blood plasma is composed by water (90%), proteins, which include albumin, globulins, fibrinogen and prothrombin (7%), organic compounds, which are basically amino acids, vitamins, hormones and lipoproteins (2%) and organic salts (1%). The RBCs are composed by proteins, where the total contents of Hb and HbO represents more than 95% of the RBC volume [34]. In terms of absorption spectrum, all blood components present smaller values than hemoglobin, with a possible exception in the deep‐ultraviolet, where albumin presents its strongest peak between 200 and 250 nm [35]. It has been reported that albumin represents 55% of the whole protein content in the blood plasma [33], but since proteins in the blood plasma represent only 7%, and considering the percentage of the blood plasma in the blood (~55%—see above), albumin represents only 2.1% of the whole blood. Since it has been reported that hematocrit (RBCs + WBCs + platelets) represents about 45% of the blood volume [33], and considering that 1% corresponds to WBCs and platelets (see above), RBCs will represent 44%. This means that the 95% of hemoglobin indicated above will correspond to a hemoglobin content of 41.8% in the whole blood. Consequently, the whole blood contains 41.8% of hemoglobin (Hb + HbO), 2.2% of RBCs membranes, 1% of WBCs and platelets, and 55% of blood plasma. Considering this difference in the concentrations of hemoglobin and albumin in the whole blood, the absorption spectrum of albumin will have a negligible contribution to the *μ*_*a*_(*λ*) of blood, even in the deep‐ultraviolet, and is not necessary for the reconstruction of the absorption spectrum of the lung tissue.

Considering now the form of the absorption spectra of blood components, a study reported some *λ* deviations between samples collected from rabbit and human blood [32]. According to the data presented in Table 1, the authors of Ref. [32] found such deviations in the peaks of the absorption bands of the following blood components: serum, plasma, Hb, albumin, RBC, lymphocyte, and platelet.

**TABLE 1:** Absorption peaks of some blood components in human and rabbit [32].

Blood component	Wavelength of absorption peak (nm)					
	Human			Rabbit		
Serum	220	300	415	210	223	278
Plasma	220	300	425		223	278
Hb	215	275	350	212	227	272
	416	540		348	416	540/578
Albumin	210	232	280	218	240	278
RBC	210	275	340	208	270	340
	416	540	575	416	543	578
Lymphocyte	215			223		
Platelet	240					

Comparing between the data presented in Table 1 for the blood components of human and rabbit, it is verified that the majority of the observed displacements occur in the deep‐ultraviolet, between 200 and 300 nm. When looking to the mean experimental *μ*_*a*_(*λ*) that was obtained for the rabbit lung (see Figure 6), it is observed that due to the broadband *λ*‐decreasing absorption of pigments, as presented in Figure 3, only the absorption bands of proteins and DNA are observed at ~230 and ~260 nm, respectively. Consequently, the use of the absorption spectrum of human hemoglobin, as presented in Figure 4C, in the reconstruction of the *μ*_*a*_(*λ*) of the rabbit lung will not introduce such *λ* displacements, and can be considered appropriate for such reconstruction. It is important to stress that after analyzing the data in Table 1, and looking at the spectrum presented in Figure 4C, that spectrum only shows the absorption bands of HbO, at 275 nm, 412 nm (Soret‐band), and 540/570 nm (*Q*‐bands). This means that for *λ*_*s*_ above ~250 nm, the magnitude of the absorption spectrum of HbO is significantly higher than the ones of the other blood components, and we can associate the spectrum presented in Figure 4C to HbO.

Considering all the absorption spectra presented in this sub‐section, a reconstruction of the mean experimental *μ*_*a*_(*λ*) of the rabbit lung was performed using Equation (1). Such digital reconstruction was optimized by selecting appropriate *p*_*i*_ values for all the tissue components, and once finished, it allowed to retrieve some physiological information about the lung. The experimental and calculated results, as well as the information retrieved from the analysis performed to the *μ*_*a*_(*λ*) of the rabbit lung and the information collected from the reconstruction are presented in Section 3.

## 3 RESULTS AND DISCUSSION

**FIGURE 5:**
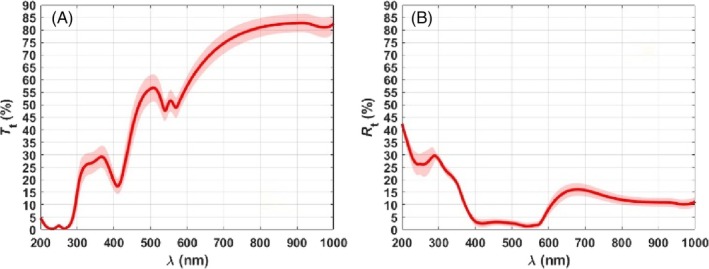
Experimental mean and SD spectra for the *T*_*t*_ (A) and *R*_*t*_ (B) of the rabbit lung.

Figure 5 presents the mean and SD for the *T*_*t*_ and *R*_*t*_ spectra that resulted from the spectral measurements with the 10 lung samples.

The mean spectra presented in Figure 5 are similar to others previously obtained for different tissues [2], and show absorption bands that correspond to proteins (230 nm) [28], DNA (260 nm) [27], hemoglobin (Soret band at 410–416 nm and *Q*‐bands at 540/570 nm) [31] and water (975–980 nm) [26].

**FIGURE 6:**
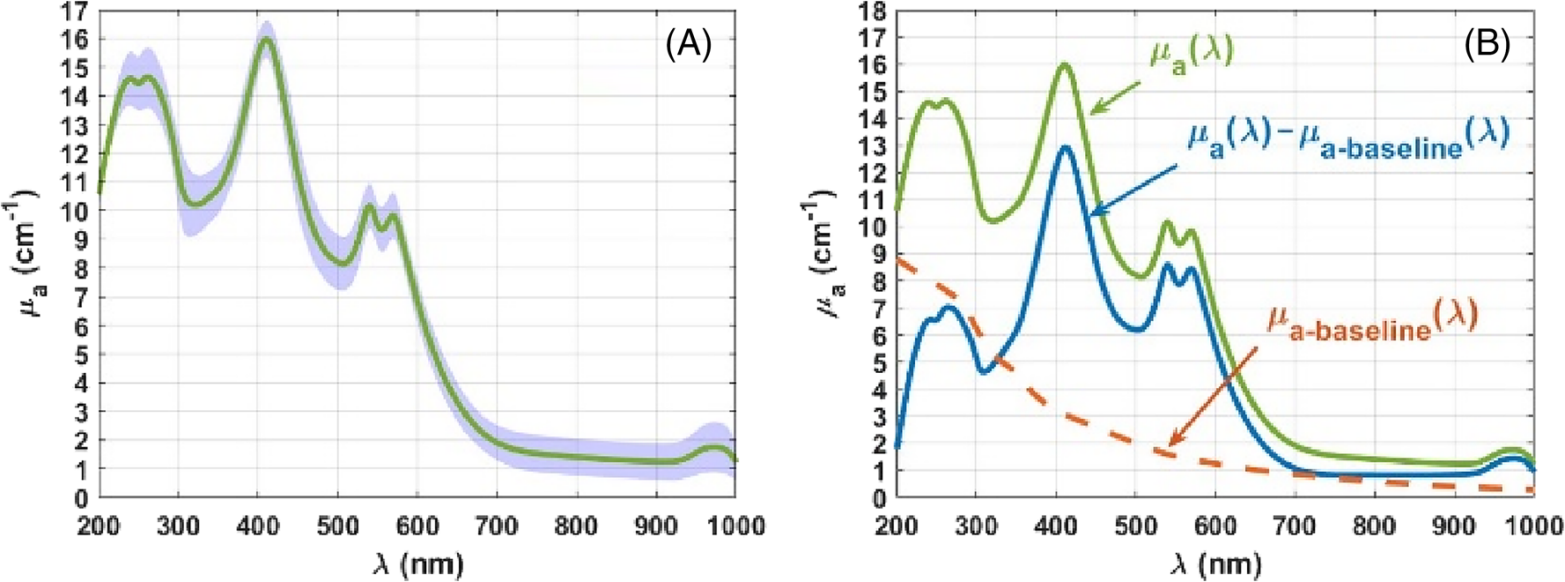
Mean and SD values for the calculated *μ*_*a*_(*λ*) of the rabbit lung (A); baseline removed spectra, *μ*_*a*_(*λ*) − *μ*_*a*‐baseline_(*λ*), of the rabbit lung (B).

Using the 10 experimental pairs of the *T*_*t*_/*R*_*t*_ spectra, calculations made with Equation (2) allowed us to obtain 10 *μ*_*a*_(*λ*) for the rabbit lung. Figure 6A presents the mean and SD values that were obtained from the 10 calculated *μ*_*a*_ spectra and Figure 6B presents the baseline removal procedure described in Section 2.3.

The mean *μ*_*a*_(*λ*) of the rabbit lung presented in Figure 6A shows an exponentially decreasing baseline as a result of the pigment accumulation and also the characteristic absorption bands of proteins, DNA, hemoglobin and water, now with central *λ*_*s*_ respectively located at 235 nm, 261 nm, 412 nm (Soret band) and 540/570 nm (*Q*‐bands), and 974 nm. With this representation, it is seen that the absorption band of proteins was right‐shifted to 235 nm, possibly due to a particular combination of the contents of the aromatic amino acids in proteins. This first analysis of the mean *μ*_*a*_(*λ*) of the lung shows that the central *λ*_*s*_ of the absorption bands of DNA, hemoglobin and water did not suffer significant shifts.

Considering the broadband *μ*_*a*‐baseline_(*λ*) seen in Figure 6A, the following step was to reconstruct it from the *μ*_*a*‐Mel_(*λ*) and *μ*_*a*‐Lip_(*λ*), so that the contributions of melanin and lipofuscin to this baseline could be retrieved and compared. Such reconstruction was optimized by considering both *p*_Mel_ and *p*_Lip_ in Equation (3) with a value of 2.85, which indicates that the accumulation of melanin and lipofuscin in the lung was made in the same proportions, only as a result of the tissue aging process, as it was previously observed for other tissues [2, 16].

Figure 6B shows the original mean *μ*_*a*_(*λ*) of the lung, the *μ*_*a*‐baseline_(*λ*) that was reconstructed with Equation (3) and the difference between these two, *μ*_*a*_(*λ*) − *μ*_*a*‐baseline_(*λ*). After removing the baseline, it is seen that the corrected absorption spectrum of the lung presents a flat baseline, with a minimal value of 0.813 cm^−1^, which is observed for the *μ*_*a*_(*λ*) − *μ*_*a*‐baseline_(*λ*) in the range between 800 and 850 nm. Considering both the *μ*_*a*_(*λ*) and the *μ*_*a*_(*λ*) − *μ*_*a*‐baseline_(*λ*) of the lung, the absorption fold‐ratios at the major absorption bands were calculated to demonstrate that the broadband absorption of the pigments hides the true fold‐ratio values of the chromophores in the lung. Such fold‐ratios were calculated by dividing the *μ*_*a*_ value observed at each absorption peak and the value of the baseline at the same *λ*. In the case of the original *μ*_*a*_(*λ*), the baseline is the one presented as a dashed line in Figure 6B and in the case of the *μ*_*a*_(*λ*) − *μ*_*a*‐baseline_(*λ*), the baseline is a horizontal line with a *μ*_*a*_ = 0.813 cm^−1^, as previously indicated. Table 2 presents those absorption fold‐ratios, before and after removing the baseline.

**TABLE 2:** Absorption fold‐ratios of major chromophores in the lung, before and after baseline removal.

Absorption bands of main chromophores	Absorption fold‐ratios	
	Original *μ*_*a*_(*λ*)	After baseline removal
Proteins (235 nm)	1.82	8.07
DNA (261 nm)	1.91	8.60
HbO Soret band (412 nm)	5.25	15.89
HbO *Q*‐band (540 nm)	6.41	10.55
HbO *Q*‐band (570 nm)	7.04	10.38
Water (974 nm)	5.62	1.77

Analyzing the values in Table 2, it is seen that by removing the baseline from the *μ*_*a*_(*λ*) of the rabbit lung, the absorption fold‐ratios at the main absorption bands increase significantly. The exception to these variations is seen for water since its absorption band is located in a spectral range where the contributions of melanin and lipofuscin are very small. Globally, the data in Table 2 shows that the broadband absorption of the pigments hides the true contents of other chromophores in the lung, such as proteins, DNA, and hemoglobin. Considering the absorption fold‐ratios obtained for DNA and hemoglobin after the removal of the baseline, they are comparable to the ones obtained in previous studies with other tissues [2, 15, 16, 17, 19]. The absorption fold‐ratio obtained for proteins was not previously reported for the lung or for other tissues, but by comparing its value in Table 2 with the one obtained for DNA, it is seen that they are similar, before and after baseline removal.

**FIGURE 7:**
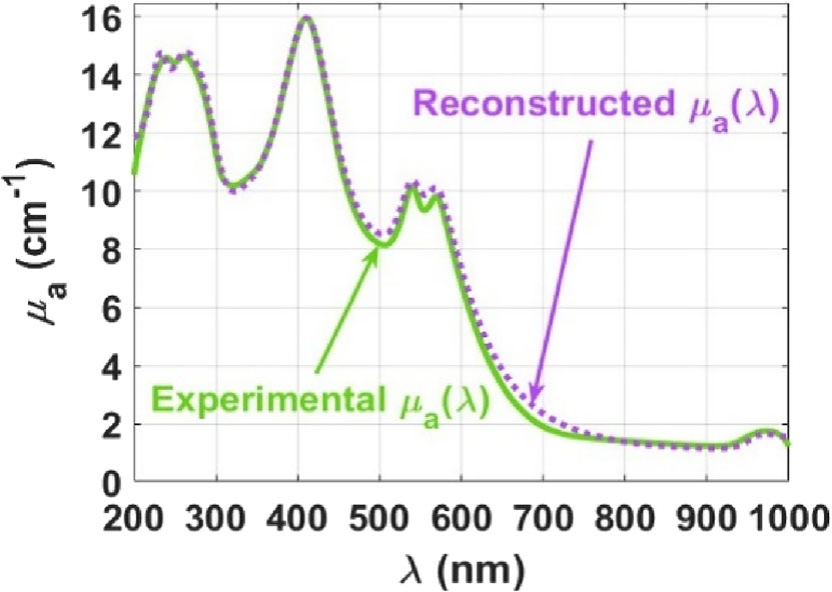
Mean experimental and reconstructed *μ*_*a*_ spectra for the rabbit lung.

To finalize this study and to pursue further physiological information, Equation (1) was used to reconstruct the mean *μ*_*a*_(*λ*) of the lung from the absorption spectra of tissue chromophores. Since the broadband absorption of the pigments was previously removed, the reconstruction of the mean *μ*_*a*_(*λ*) of the lung was made in two steps. First, the *μ*_*a*_(*λ*) − *μ*_*a*‐baseline_(*λ*) that is presented in Figure 6B was reconstructed with Equation (1), using the absorption spectra presented in Figure 4. Finally, the *μ*_*a*‐baseline_(*λ*) that was previously obtained was added to the reconstructed spectrum to compare it with the mean experimental *μ*_*a*_(*λ*) of the lung. Figure 7 presents both the mean experimental and the reconstructed *μ*_*a*_(*λ*) for comparison.

From Figure 7, we observe a significantly good matching between the mean experimental and the reconstructed *μ*_*a*_(*λ*) of the lung. This result shows that the selection of the tissue components and the *p*_*i*_ values to use in Equation (1) to obtain the reconstructed spectrum was appropriate.

The most significant difference found between the two spectra regards the hemoglobin component, since the reconstructed spectrum is a little higher than the experimental spectrum between approximately 450 and 750 nm. Since the absorption spectrum of hemoglobin that was used in the reconstruction was measured in this study from a blood dilution in water, the observed mismatch shows that the concentration of blood used in such dilution might not be exactly the one that corresponds to the blood content in the rabbit lung. Regardless of such difference, considering the entire spectral range, the reconstructed spectrum is in good agreement with the mean experimental *μ*_*a*_(*λ*) of the lung.

The water content in tissues is known to vary within a wide range, but for lung, a content of 80% is a good estimate [36, 37]. Considering this value for the water content in the rabbit lung, the remaining 20% correspond to the combination of the other chromophores that were identified in our analysis of the mean experimental *μ*_*a*_(*λ*) of the lung: melanin, lipofuscin, DNA, proteins, and hemoglobin. This means that a conversion of the obtained *p*_*i*_ values to a scale between 0% and 20% allows a better estimation for the concentrations of those chromophores in the rabbit lung. Such conversion was performed and Table 3 presents both sets of data: the *p*_*i*_ values obtained in the reconstruction of the mean experimental *μ*_*a*_(*λ*) and the calculated concentrations of chromophores with the corresponding SD values.

**TABLE 3:** Weights of the tissue chromophores used in the reconstruction of the *μ*_*a*_(*λ*) of the rabbit lung and calculated concentrations.

Biological component/chromophore	Weight—*p*_*i*_	Concentration ± SD (%)
Water	0.67	80.00
Melanin	2.85	2.30 ± 0.09
Lipofuscin	2.85	2.30 ± 0.09
DNA	3.53	2.85 ± 0.63
Proteins	2.90	2.35 ± 0.24
Hemoglobin	12.62	10.20 ± 2.15

In a first analysis of the *p*_*i*_ values in Table 3, we see that, although hemoglobin has the highest value, no direct information about the chromophores' concentrations can be retrieved. Considering the calculated concentrations, it is confirmed that, apart from water, hemoglobin presents the highest concentration in the rabbit lung. The calculated concentrations for the other chromophores are all in the same range, which is about 5× less than hemoglobin.

The SD values that were calculated for the concentrations of the lung's chromophores were obtained based on the spectral SD that is presented in Figure 6A. The first step to obtain these values was to add the spectral SD to the mean experimental *μ*_*a*_(*λ*) of the lung that is presented in Figure 7. Such spectrum was reconstructed as before, now leading to new *p*_*i*_ values, which were once again converted into concentrations, considering a concentration of 80% for water in the lung, as before. The difference between the new concentrations and the ones obtained with the reconstruction of the mean experimental *μ*_*a*_(*λ*) of the lung correspond to the SD values presented in the last column of Table 3. According to these estimated SD values, we verify that both melanin and lipofuscin present the smaller values, which indicates that variations in the concentrations of pigments should be smaller than 0.1% to match their concentration in all samples studied. DNA and proteins present higher SD values than the pigments, but lower than 1% in both cases. This means that the maximum variation of proteins and DNA in all samples studied is very small. In opposition, the SD value obtained for the concentration of HbO is the highest—2.15%. This result indicates that the blood content in the samples used in the study has a higher variation than the contents of the other lung components.

To discuss the estimated concentration of hemoglobin, we consider a previous study that reported the blood volume concentration (BVC) in human lungs in vivo, both for healthy and for patients with systemic sclerosis [38]. The BVC values reported in that study were 20 ± 5% for the healthy and 16 ± 4% for the diseased lungs. Considering that BVC might be similar to that for rabbit lungs in vivo, we took as reference the 20% value reported for the BVC in human healthy lung in vivo. As previously indicated, it is known that blood contains various components itself, such as hemoglobin and blood plasma, which volume ratio is associated with hematocrit. In this way, the blood volume in lungs can be reconstructed from the concentration of hemoglobin presented in Table 3. In the case of adult rabbits, it has been reported that the hematocrit in the blood can range from 33% to 50% [39]. If we consider an average value of 42% for the hematocrit, then the volume of RBCs can be estimated as 41%, because approximately 1% is occupied by the other blood cells. Due to the 95% of hemoglobin content in RBCs, then in any blood volume we will have 39% of hemoglobin, 2% of RBC membranes, 1% of the rest of the cells and 58% of blood plasma. Using, from Table 3, the experimental value for the hemoglobin content in the lung tissue as 10.2%, we can recalculate it to the blood volume basing on the above estimates. In such calculation hemoglobin amounts to 39% of the BVC and 61% is the rest of blood components, which percentage in the lung can be obtained from the proportion (61% × 10.2%)/39% = 15.9%, that is, BVC = 10.2% + 15.9% = 26.1%. This value is well fitted to that measured in Ref. [38] as 20 ± 5%. The data used in this discussion that was retrieved from Ref. [38] corresponds to the in vivo situation, where the lungs are filled by air, thus lung volume should be somewhat higher than in the ex vivo situation that corresponds to the present study. If this is the case, then our estimate of 26.1% for the blood volume in the rabbit's lungs looks very realistic. The 15.9% portion of blood contains plasma, RBC membranes, and the rest of cells. Using the similar calculations, the cellular part of blood can be estimated as 0.8% of lung volume, thus plasma portion is of 15.1% and it is a part of the total water content, which we considered as 80% for the lung of adult rabbits. This means that if we retrieve the blood plasma volume from the water volume, we get the water content of 64.9% that corresponds to the water contained in other lung components. This value is more realistic for collagenous structures (including all types of water‐binding states) like for example bloodless dermis, which is 65% [40, 41]. Although we have obtained a good matching between the reconstructed and the mean experimental *μ*_*a*_(*λ*) of the rabbit lung in this study, and good estimates for the contents of the lung chromophores, we see that such physiological information was only possible through a scale conversion of the *p*_*i*_ values that were used, which was based on the published concentration of water in the lung. The necessity of performing such scale conversion and using normalized *μ*_*ai*_(*λ*) for the various chromophores in the lung was due to the significant difference in magnitudes found in the original *μ*_*ai*_(*λ*) that were obtained from different sources. This means that the method used in this study can be applied to study the concentrations of chromophores in other tissues, provided that the water concentration in those tissues is known.

## 4 CONCLUSION

The present study showed that the broadband absorption coefficient spectrum of any biological tissue can be reconstructed by a weighted combination of the absorption spectra of the components that such tissue contains. By performing such a combination for the rabbit lung, it was possible to identify that this tissue is composed of proteins, DNA, hemoglobin, and water. A first analysis made to the mean experimental *μ*_*a*_(*λ*) of the lung indicated the accumulation of pigments in the lung through the observation of an exponentially decreasing baseline. It was possible to reconstruct this baseline by combining the absorption spectra of melanin and lipofuscin and to verify that such accumulation was similar for both pigments as a result of the aging process of the lung tissues analyzed. After removing this baseline, the calculation of the absorption fold‐ratio for proteins was made for the first time, and the calculation of the absorption fold‐ratios for DNA and hemoglobin showed similar values to those previously observed for other tissues. By reconstructing the complete mean experimental *μ*_*a*_(*λ*) of the lung as a weighted combination of the absorption spectra of its various chromophores, it was possible to obtain a good matching between the reconstructed and experimental spectra, and also to quantify the concentrations of the chromophores. Such concentrations were obtained only after a scale conversion of the *p*_i_ values that were used in the spectral reconstruction, but provide valuable exploratory information for the ex vivo rabbit lung. Analyzing such data, it was observed that by considering that the 80% of the lung volume is water, the second major component is hemoglobin, which presents a concentration about 5× higher than each of the other components. Similar studies can be performed with this reconstruction method to other tissues, allowing to quantify the concentrations of chromophores. For tissues where the water concentration is unknown, additional experimental measurements to evaluate such value are necessary. We plan to use this method to study the concentrations of chromophores in other tissues. Additionally, we plan also to perform noninvasive *R*_*d*_ spectroscopy measurements from in vivo tissues, which combined with ML algorithms can be used to reconstruct their broadband *μ*_*a*_(*λ*). By performing such research, the use of the same reconstruction method may allow to provide in vivo diagnostic information.

## AUTHOR CONTRIBUTIONS

M.R.P. was involved in the investigation and writing—original draft. V.V.T. and L.M.O. were involved in conceptualization, investigation, writing—review and editing.

## CONFLICT OF INTEREST STATEMENT

The authors declare that there is no conflict of interest.

## Data Availability

The data that support the findings of this study are available from the corresponding author upon reasonable request.
